# Supplementary material for: Neural signatures of automatic letter–speech sound integration in literate adults
Source: Imaging Neurosci (Camb). 2025 Nov 20;3:IMAG.a.1021. doi: 10.1162/IMAG.a.1021 (PMC12635484; doi:10.1162/IMAG.a.1021)
Supplement: Supplementary Material [file IMAG.a.1021_supp.pdf]

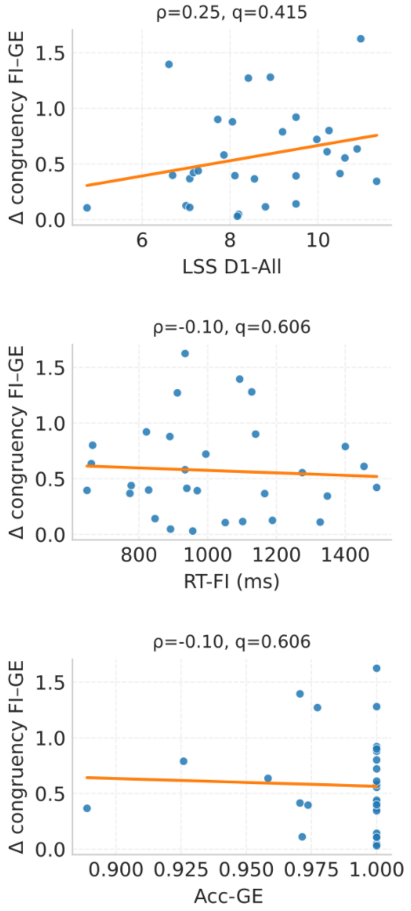

Supplementary Figure S1. Correlations between the neural signature of automaticity and behavioral performance. Scatter plots illustrate the relationship between the neural signature of automaticity (y-axis) and various behavioral measures (x-axes). The neural signature is defined as the magnitude of the difference in the LSS congruency effect between overlearned (Finnish) and newly learned (Georgian) pairs, extracted from the left medial parietal cortex (Δ congruency FI–GE). Behavioral variables include individual LSS learning speed, which was defined as the learning index on Day 1, computed for all stimuli (LSS D1-All) and separately for Learnable (LSS D1-Learnable) and Control (LSS D1-Control) pairs following our previous study (Xu et al., 2020), and in-MEG task reaction time (RT) and accuracy (Acc) for both overlearned (FI) and newly learned (GE) pairs in the current study. Each panel displays the individual participant data points, the linear fit (orange line), the Spearman's rho (ρ), and the FDR-corrected p-value (q). No correlations reached statistical significance after correction for multiple comparisons.
